# Amenorrhea

**Published:** 1854-01

**Authors:** 


*Amenorrhea.*—Dr. Plumer, of Richmond, Va., reports the successful treatment of Amenorrhea with half-ounce doses of the liq. acet. ammonia, properly prepared with dilute acetic acid.—*From the N. H. Journal of Medicine.*

